# Digital sustainability: basic conditions for sustainable digital artifacts and their ecosystems

**DOI:** 10.1007/s11625-016-0412-2

**Published:** 2016-12-01

**Authors:** Matthias Stuermer, Gabriel Abu-Tayeh, Thomas Myrach

**Affiliations:** University of Bern, Bern, Switzerland

**Keywords:** Digital sustainability, Sustainable development, Knowledge commons, Linux, Bitcoin, Wikipedia, Linked Open Data

## Abstract

The modern age has heralded a shift from the industrial society, in which natural resources are crucial input factors for the economy, towards a knowledge society. To date, sustainability literature has treated knowledge—and in particular digital artifacts—mainly as a means to the end of achieving sustainable development. In this conceptual paper, we argue that digital artifacts themselves ought also to be considered as resources, which also need to be sustainable. While over-consumption is a problem facing natural resources, with sustainable digital artifacts, underproduction, and underuse are the biggest challenges. In our view, the sustainability of digital artifacts improves their potential impact on sustainable development. A theoretical foundation for digital artifacts and their ecosystem allows us to present the relevant research on digital information, knowledge management, digital goods, and innovation literature. Based on these insights, we propose ten basic conditions for sustainable digital artifacts and their ecosystem to ensure that they provide the greatest possible benefit for sustainable development. We then apply those characteristics to four exemplary cases: Linux kernel development, Bitcoin cryptocurrency, the Wikipedia project, and the Linking Open Drug Data repositories. The paper concludes with a research agenda identifying topics for sustainability scholars and information systems academics, as well as practitioners. A number of suggestions for future studies on digital sustainability are also put forward.

## Introduction

The Brundtland Report provides the most popular definition of sustainable development: “development that meets the needs of the present without compromising the ability of future generations to meet their own needs” (World Commission on Environment and Development 1987, p. 37). In that report, knowledge and technology are addressed as means of supporting sustainable development. However, the specific role of knowledge and its use remain somewhat non-specific. Miller et al. (2014), for example, criticized that many scholars remain rather vague in demanding a further accumulation of knowledge to cope with environmental and societal issues. With respect to this critique, solution-based ideas are needed in the context of the ways in which knowledge is accumulated, made accessible, and exploited. Knowledge and the impact of technology on the creation and use of such knowledge could be considered as a vehicle to support sustainable development (Melville 2010; Elliot 2011; Seele 2014). In our view, knowledge has to be seen as a resource that itself should be sustainable, to preserve its value for society and ensure that it can permanently contribute to the goals of sustainable development.

With the ever-increasing use of computer infrastructures, a growing proportion of recorded information has become digital. It is estimated that in 1993, only 3% of the world’s recorded information was stored digitally; this figure had increased to approximately to 94% by 2007 (Hilbert and Lopez 2011). We define digital artifacts as entities that consist of strings of 0 and 1, which can be interpreted by technical devices, like computers, to provide some meaning. Thus, digital artifacts have become the basic incarnations of knowledge in our times. These digital artifacts have a number of specific characteristics resulting in various benefits and downsides compared to traditional media. In particular, digital artifacts are not self-contained and are embedded in wider, constantly changing ecosystems (Kallinikos et al. 2013). This means that digital artifacts are influenced not only by the technical components, but also the social ecosystem of people and institutions, through their acts of creation and use. Individuals and organizations are crucial for digital artifacts, since they artificially create digital artifacts in the first place and the sole purpose of the existence of digital artifacts is for these to be used by other individuals and organizations. Our question in this context is: *What are the basic conditions for digital artifacts and their ecosystems that need to be fulfilled in order for them to be constantly created and used, thus providing the greatest possible benefit to sustainable development?*

Our paper is structured as follows: First, we explain the theoretical basis, covering the characteristics of digital artifacts and their relevant ecosystems. Furthermore, we explain an important difference between natural resources and digital artifacts with respect to creation and use. Next, we develop a concept for the sustainability of digital artifacts and their ecosystem by proposing ten specific basic conditions for digital artifacts, their surrounding ecosystems, and their contribution to sustainable development. We then illustrate our concept by analyzing four initiatives and their resulting digital artifacts in terms of the proposed basic conditions. This enables us to draw conclusions as to the extent to which the discussed digital artifacts and their ecosystems may be perceived as sustainable and contribute to sustainable development. Finally, we draw conclusions, explain the limitations of our framework and provide indications for further research topics related to the concept of digital sustainability.

## Theoretical foundation of the sustainability of digital artifacts and their ecosystem

Thinking of the sustainability of digital artifacts and their ecosystems as we understand it touches on several different research domains. The following literature-based analysis is centered on the digital artifact and the ecosystem in which it is embedded. For each of these two concepts, we establish important characteristics and describe in more detail how these two concepts relate to one another. This provides the theoretical foundation of the basic conditions for the sustainability of digital artifacts and their ecosystem, as explained in “Cases of sustainable digital artifacts and ecosystems”.

### Digital artifacts

The rise in the use of computers has led to a profound change in the nature of records and record-keeping. Because the predominant paradigm of electronic data processing is digital, the representation of data to be processed by computers also had to be made digital. Digital data is stored in computer files. The various programs installed on computers determine what they do with data and the specific problem domain in which the data are employed. Computer programs consist of code, which tells the computer how data is to be processed by the machine. Technically, computer code is also data, which is recorded in computer files. Both data files (texts, pictures, audios and videos) and computer code files (machine code and source code) can be subsumed under digital artifacts (Kallinikos et al. 2013).

A remarkable characteristic of digital artifacts is that they are not self-contained. First of all, a technical device is needed to process a digital artifact. Second, digital artifacts depend on other digital artifacts. To read a digital data file, for instance, an application system is needed (which consists of at least one executable program file) and to access the data file on the storage media, the functions of an operating system (which typically consists of several executable program files) also need to be used. Thus, any digital artifact is embedded in a wider and constantly shifting ecosystem (Kallinikos et al. 2013). In a narrow interpretation, a digital ecosystem consists of all hardware devices, program files, and data files that the user needs to process data. In a wider sense, the ecosystem also comprises the social elements which lead to the creation and use of digital artifacts (Faulkner and Runde 2013).

Digital artifacts are quite often distinguished from physical or material objects and characterized as intangible or virtual objects, but they may be considered to be both at the same time (Leonardi 2010; Blanchette 2011). On the one hand, every digital artifact at any time of its existence is represented as an ordered form of physical impulses, bound to hardware devices like computers, storage devices, networks, etc. The files occupy physical space. If computer files are stored, the capacity of the storing device is limited. In the same manner, sending a digital file over a network is limited by its bandwidth. On the other hand, digital artifacts appear to the user in a virtual form created by the processing application software. Thus, e.g. the paper-like presentation of a text file (“what you see is what you get”) is the product of the text processor, which emulates the appearance of a printout.

Digital artifacts have some distinct characteristics that distinguish them from traditional non-digital records (e.g. Kallinikos et al. 2013). We consider two properties to be particularly important: first, digital artifacts can be replicated easily (reproducibility). As a consequence, digital content may be much more easily distributed than any other content on traditional media (Benkler 2006; Kallinikos et al. 2013). Second, digital artifacts can be edited and, therefore, changed (transmutability) (Kallinikos et al. 2013). This provides enormous flexibility in working with digital content, adapting any given content and reusing contents in another context.

With regard to the preservation of recorded information, the effect of digitizing is ambiguous. On the one hand, the use of digital artifacts is not subject to abrasion. Regardless of how often a digital artifact is used, it retains the exact same quality. Choi et al. (1997) also refer to this characteristic as ‘indestructibility’. As mentioned above, digital artifacts physically exist at any time in data processing devices. Thus, the media on which data are stored may be damaged and technical malfunction is always a possibility. There may also be organizational reasons for data loss. On account of properties like reproducibility and transmutability, digital artifacts are quite volatile and perhaps somewhat abstract in people’s minds. This could lead to careless behavior towards data artifacts (Ponemon 2013).

It has been established that any digital artifact is embedded in a wider (technical) ecosystem. In consequence, its use depends on the existence of the various elements of this ecosystem. Thus, technical obsolescence due to changing technical equipment poses a major threat for the long term preservation of data (Rothenberg 1999). This may apply to the obsolescence of the media: the medium disappears from the market, appropriate drives capable of reading the medium are no longer produced, and media-accessing programs capable of controlling the drives and deciphering the encoding used on the medium are no longer available for new computers. Data are inherently software-dependent and can only be interpreted by a computer program. Application programs can also become obsolete. To keep these programs running, the proper operating system environment is needed. Operating systems are bound to specific computer hardware, which itself becomes obsolete relatively quickly. Subsequently, all the digital artifacts affected would be rendered obsolete, even though they might physically be retained. Protecting digital artifacts against these various threats demands an awareness of potential threats and constant efforts to maintain the value of the stored data.

### The ecosystem

In our remarks on digital artifacts and their characteristics, we established that any digital artifact is embedded in, and depends on, a wider ecosystem. Pursuant to a narrow technical interpretation, a digital ecosystem consists of all hardware devices, program files and data files that the user needs in order to process data. Information systems, however, may be interpreted as socio-technical systems in which human actors and technical components are related and interact with one another (Bostrom and Heinen 1977; Ropohl 1999; Mumford 2006). Thus, in a wider sense, the digital ecosystem involves not only the technical components, but also the social elements. We characterize the relationship between the digital artifacts and their social ecosystem as acts of creation and use of digital artifacts. While digital artifacts represent recorded information, the surrounding ecosystem of individuals and organizations (Messerschmitt and Szyperski 2005; Bosch 2009; Kallinikos et al. 2013) holds know-how and experience related to the creation and use of a digital artifact. To obtain a deeper insight into important principles governing the behavior of the social ecosystem towards the creation and use of digital artifacts, we will now explore the domains of knowledge management and digital goods.

### Knowledge management

With respect to knowledge, it is important to distinguish between tacit knowledge and explicit knowledge (Nonaka 1994; Polanyi 1967). Explicit knowledge is expressed in some form of record (digital artifact). Tacit knowledge exists in the brains of people and consists of cognitive (e.g. mental models) and technical elements (e.g. know-how/skills), which are sometimes hard to formalize and communicate because they are rooted in a specific context. There are different forms of transformation of knowledge between persons (Nonaka and Konno 1998): the transformation between tacit and explicit knowledge is handled by externalization (tacit to explicit) and internalization (explicit to tacit), while the transfer of tacit knowledge is achieved through socialization (tacit to tacit). Regardless of these transformations, ultimately, knowledge must be anchored in individuals’ brains, thus making it tacit knowledge.

Wenger (2004) noted that knowledge is based not only on individuals, but also on the community of practice to which individuals belong, which helps them decide what is right and wrong. He believes that knowledge is linked to the community of practitioners: “Communities of practice are groups of people who share a passion for something that they know how to do, and who interact regularly in order to learn how to do it better” (Wenger 2004, p. 2). Only within the community of practice do people understand the difficulties and insights associated with explicit knowledge (represented as digital artifacts) to a sufficient degree to improve learning. For a community of practice to prosper, knowledge cannot be hoarded; sharing and stewarding of knowledge can be applied by other practitioners, allowing them to increase the performance of the entire community. Thus, shared tacit knowledge (either by socialization or externalization) is important for using knowledge in a group to achieve certain goals. However, the sharing of tacit knowledge is not per se sufficient to establish a fruitful cooperation. In addition, a participatory culture is needed so that productive ecosystems can be attained (Wenger 2004).

### Economics of digital goods

Digital goods “are bitstrings, sequences of 0 and 1 s, that have economic value” (Quah 2003). The difference in the definition from digital artifacts lies in the economic value. The economic value of goods stems from the fact that they serve as a means of satisfying a need or a desire. In the economy, people usually have to pay for the goods because the producers demand a price in return for their efforts. Because digital artifacts can be replicated easily, the reproduction of a digital artifact results in marginal costs only (Faulkner and Runde 2013; Rifkin 2014). Therefore, digital records can be distributed easily. Furthermore, digital artifacts are characterized as being non-rival, among other things (Quah 2003; Hess and Ostrom 2006; Baldwin and Clark 2006; Wasko et al. 2009). This means that the use of these artifacts by other people usually does not impair their own use. As a result, they are more inclined to share their digital artifacts with others (Benkler 2006). Because individuals cannot be effectively excluded from using digital artifacts and the use by one individual does not necessarily exclude another person from using them, Kogut and Metiu (2001) claim that, in fact, digital artifacts have the basic properties of a common-pool resource. Thus, it might be difficult to convince people to pay some price for these products as the effort involved in distribution results only in marginal costs.

Contrary to the reproduction of digital artifacts, the development of digital artifacts is not without cost. The question, therefore, is under which circumstances people are motivated to develop these resources. In their work, von Hippel and von Krogh (2003) analyzed two commonly known models for innovation: the private model (Arrow 1962; Dam 1995) and the collective action model (Hardin 1982). The private model of innovation is driven by the incentive of intellectual property rights of firms. In return for being innovative, firms can protect their property with copyrights and patents, thus dictating the licensing costs or the selling price of their products. The benefit of this model is that there is a strong incentive for innovation. The downside is a loss of societal knowledge. This relative loss of knowledge occurs because the amount of absolute knowledge in society remains constant if an innovative firm is able to enlarge its knowledge but does not make that knowledge available to society. In the collective action model, innovation is provided as a public good. The benefit of this model is that society does not experience any loss in knowledge, neither absolutely nor relatively. The downside is that there are less extrinsic incentives for innovation. This may lead to a collective action problem, since those with extrinsic motivations are unlikely to want to take responsibility for the creation and maintenance of the public good. However, there are several papers that show that there may be sufficiently high numbers of individuals with intrinsic motivation, circumventing the collective action problem (Malone et al. 2010; von Krogh et al. 2012).

As the analysis of the two innovation models reveals, the two models have opposing benefits and downsides relating both to the production side (creating and maintaining innovative goods) and to the user side (availability of societal knowledge). There may be some ways of potentially overcoming these trade-off problems: one rather traditional argument for the provision of public goods is that the state should provide them, rendering the collective action problem irrelevant. With respect to non-state activities, von Hippel and von Krogh (2003) propose a private-collective innovation model, which can be seen as a combination of both other models. The private-collective innovation model assumes the development of common-pool resources, as in the collective action model. To overcome the downside of the lack of innovation, it is assumed that there are incentives for firms and individuals to develop common-pool resources without being incentivized by property rights. Stuermer et al. (2009) list some of these possible incentives: low knowledge protection costs, learning effects, reputation gain, adoption of innovation, increased innovation at lower costs, lower manufacturing costs, and faster time-to-market. This approach demands business models that combine open licensing regimes with services that generate revenues for the participating companies.

### Creation and use in the natural and the digital world

The specific characteristics of digital artifacts and their surrounding ecosystems outlined above have significant implications for the creation and use of digital artifacts. In order to better understand these implications, we define the difference between natural resources and digital artifacts. It is important to highlight two dimensions: on the one hand, the creation and improvement of the artifacts and on the other hand, their use and sharing. Natural resources already exist in nature, whereas digital artifacts have to be created by humans and machines. Individual or organizational effort is necessary to create digital artifacts. However, the use of digital artifacts does not diminish their value. On the contrary, the value to society as a whole increases the more people have access to its use. In contrast, the use of natural resources needs to be regulated in order to reduce consumption of non-renewable resources and prevent the over-consumption of renewable resources (Wackernagel and Rees 1997; Porritt 2007).

Distinguishing between the two dimensions of creation and use leads to the conclusion that a sustainable development of natural resources (environmental sustainability) is critical with respect to the use-dimension, whereas sustainable development of digital artifacts (sustainability of digital artifacts and their ecosystem) is critical with respect to the creation-dimension. Table 1 summarizes this conclusion.

**Table 1:**
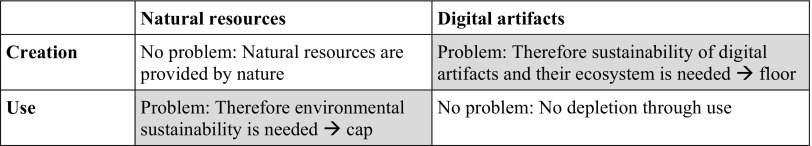
Creation and use dimension of natural resources and digital artifacts

Adopting the concept of the carrying capacity model (Wackernagel and Rees 1997), we conclude that the limitation of the use of natural resources is the “cap”, while the need for favorable basic conditions for the creation of digital artifacts may be called the “floor”. Thus, the carrying capacity model limits the use of natural resources with a “cap” (carrying capacity), while the “floor” model constitutes an inverse carrying capacity model for a successful dissemination of knowledge. With respect to sustainability, over-consumption is a problem with natural resources, while under-production is the challenge with digital artifacts.

Because the use of digital artifacts produces value but no deterioration, it appears desirable from the societal perspective that digital artifacts, which potentially have a positive impact on sustainable development are used as much as possible. This is an inversion of the situation with natural resources, which are limited and, therefore, should not be exploited excessively. There may be several reasons why digital artifacts are not exploited to their full potential. Individuals or organizations may not be aware that relevant knowledge exists or are unaware of where or how to find it. Furthermore, man-made barriers such as intellectual property rights may restrict access to knowledge (Shapiro 2001). In addition, knowledge recorded as digital information can also become inaccessible due to technical obsolescence (Smith Rumsey 2010). All of these reasons may cause knowledge to become unsustainable when underused. In our view, *the sustainability of digital artifacts and their ecosystem is achieved by producing, developing, maintaining and ensuring access to digital artifacts in a way that ensures their creation and facilitates their use.* This allows the potential of knowledge for achieving goals of sustainable development to be exploited to the fullest.

## Basic conditions for the sustainability of digital artifacts and their ecosystem

In this chapter, we propose ten basic conditions that build a foundation for achieving sustainability of digital artifacts and their ecosystem. According to our distinction between the digital artifacts themselves, the surrounding ecosystem in which they are embedded, and the position of the ecosystem in the whole world, we assign each of these conditions to one of the three concepts. The first group of basic conditions can be considered to be content-specific properties. They cover explicit knowledge e.g. in the form of source code, data or multimedia content. The conditions elaborateness, semantic data, transparent structures, as well as distributed location, describe the substance of the digital artifact. The next group relates to social structures defining rules and norms of individuals and organizations, the way in which they can/are permitted to be used and contribute to digital artifacts. These five basic conditions all pertain to the surrounding community in regard to legal requirements, knowledge creation, organizational and financial management: an open licensing regime, shared tacit knowledge, participatory culture, good governance, and diversified funding. The last basic condition refers to the contribution to sustainable development, which should be positive. All ten basic conditions together result in sustainable digital artifacts.

**Fig. 1:**
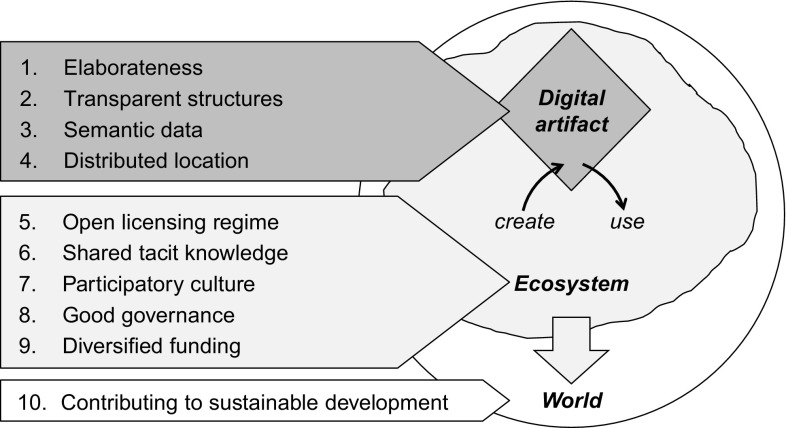
Basic conditions of sustainable digital artifacts

Figure 1 shows the proposed basic conditions that govern the creation and use of digital artifacts by stakeholders within the ecosystem in the world. Subsequently, each basic condition is explained and discussed in more detail. Table 2, below, provides a summary of each basic condition, indicating its benefit to a sustainable development.

**Table 2:** Basic conditions of sustainable digital artifacts

Condition	Explanation	Benefit for sustainable development	References
*Digital artifact*			
1. Elaborateness	Elaborateness of digital artifacts is determined through their modularity, integrity, accuracy, robustness, and other characteristics regarding the quality of their substance	Elaborateness of digital artifacts creates immediate value to their users by applying data or software for specific problems	Raymond (2001); Stamelos et al. (2002); Wang and Strong (1996)
2. Transparent structures	Transparent structures signify technical openness allowing access to the inner structures of digital artifacts, such as source code, standard specifications, content, or data structures	Transparent structures enable improvements and allow verification of digital artifacts, thus reducing failures and errors and increasing trust in technologies	Corrado (2005); Coyle (2002); Park and Oh (2012)
3. Semantic information	Semantic information makes complex digital artifacts more easily intelligible to humans and machines through comprehensible structures and meta data	Semantic information enables individuals, organizations and eventually society to absorb previously created knowledge and to advance that knowledge	(Edmunds and Morris (2000); Jackendoff and Jackendoff (1992); Sheth (1999)
4. Distributed location	Distributed location means data, software and other digital artifacts are stored and operated on multiple sites, e.g. through replicated data storage or peer-to-peer technology	Distributed location increases the long-term availability of digital artifacts and their operational reliability to the benefit of society	Baran (1964); Ripeanu (2001); Schollmeier (2001)
*Ecosystem*			
5. Open licensing regime	Open licensing regimes grant anyone the right to use and modify digital artifacts at no cost and for any purpose, thus providing improvements and enhancements without limitations	Open licensing regimes allow society to fully exploit the intellectual capacity of humanity e.g. for the solution of problems and for increases in prosperity	Scacchi and Alspaugh (2012); Shapiro (2001)
6. Shared tacit knowledge	Shared tacit knowledge of digital artifacts means there are many individuals and organizations that know through their experience how to understand, use, and modify the digital artifacts	Shared tacit knowledge reduces dependence of society on a single or a few individuals, corporations or other organizations. Thus, it empowers individuals and organizations to contribute to digital artifacts, increasing their elaborateness and longevity through future adaptations	Nonaka and Konno (1998); Wenger (2004); Benkler et al. (2015)
7. Participatory culture	Participatory culture signifies permeability of contributions throughout the entire lifecycle of digital artifacts, enabling peer-review processes on all levels	Participatory culture allows the creation of active ecosystems surrounding digital artifacts, bringing together knowledge and experience of all contributors	Lakhani and Von Hippel (2003); Benkler et al. (2015)
8. Good governance	Good governance means the digital artifact and its ecosystem is not controlled by a single individual or organization, but governed decentralized by its contributors and other stakeholders	Good governance reduces dependency on a single entity, thus preventing abuse of the digital artifact by self-serving commercial or other interests to the disadvantage of society	Ostrom (2005); O’Mahony and Ferraro (2007); Viégas et al. (2007)
9. Diversified funding	Diversified funding allows cost covering of infrastructures, contributions, and other spending from various financial sources	Diversified funding reduces control of financial resources by a single entity, thus increasing the independence of future improvements and decreasing the risk of conflicting interests	Riehle (2010); Baars and Jansen (2012); Belleflamme et al. (2014)
*World*			
10. Contribution to sustainable development	Contribution to sustainable development means sustainable digital artifacts must provide positive ecological, social or economic effects	Contribution to sustainable development aligns the use of digital artifacts with the global goals of sustainable development	Kossahl et al. (2012)

### Elaborateness

In discussing the theoretical foundations of digital artifacts above, we characterized the transmutability (editable and reprogrammable) of digital artifacts as an important property. Even though every digital artifact may, in principle, be edited or reprogrammed, it is important how easily this can be done. In order to continuously enhance a digital artifact and to obtain reliable information from it, its content and structure need to be well elaborated from the start. The quality of the data or software, defined by properties like correctness, modularity, integrity, accuracy, robustness, and other characteristics (Stamelos et al. 2002; Wang and Strong 1996), is essential for the sustainable enhancement of a digital artifact. However, while the initial scope of the digital artifact has to meet the level of a “plausible promise” (Raymond 2001), it does not need to be complete in its functionality or data set. In an ideal world, a vibrant ecosystem of a sustainable digital artifact is capable of enhancing and adapting the artifact continuously. How such processes succeed in detail is the subject of many current studies (e.g. Ekbia 2009). Benkler et al. (2015) have found that the quality of digital artifacts is one of the key areas of ongoing research within peer-production ecosystems.

### Transparent structures

Both documents and software are often encoded in machine-readable data formats such as binary files. These types of digital artifacts are not comprehensible for humans and thus cannot be corrected or enhanced (Bradley 2007). In order to benefit from the transmutability of digital artifacts (and, therefore, the possibility to use them in another context) transparent structures are required. Transparent structures lead to technical openness in the form of the detailed specification of data structure and formats, openly accessible source code of software, or freely available information architecture and documentation (Corrado 2005; Coyle 2002; Park and Oh 2012). Such digital artifacts can be verified and improved by interested data scientists or programmers, thus reducing errors and increasing trust in technologies.

### Semantic data

As discussed in the theoretical foundations, digital artifacts represent a syntactical dimension. There are also semantics associated with the data, representing its meaning. Information on the meaning and properties of data is meta-data, i.e. data about data, or semantic data. Semantic data is necessary for the automated linking of data by software algorithms. The vast amount of digital knowledge available leads to information overload (Edmunds and Morris 2000), while meta-data allows information to be pinpointed more precisely, thus reducing information overload (Jackendoff and Jackendoff 1992; Sheth 1999). Semantic data allows large and complex digital artifacts, such as data or software components, to be found more easily and linked by humans and machines with other items of information. This facilitates the use and enhancement of such digital artifacts, allowing them to be combined logically with previously created knowledge and thus advancing that knowledge.

### Distributed location

In the theoretical foundation chapter, we asserted that digital artifacts are both immaterial and material objects. Every digital artifact is at any time always physically present, since it has a persistent location on some storage unit. Therefore, digital artifacts such as data and software are at risk of being lost as a result of data loss, hardware crashes or other accidents. Server systems may become dysfunctional when the server is hacked or disconnected due to technical problems, for example. A decentralized architecture through the distributed location of the digital artifacts decreases the vulnerability of the network (Baran 1964) and thus increases the long-term availability of data and software. Peer-to-peer technology presents an ideal approach of redundancy on different locations reducing data loss and system failure to a minimum (Ripeanu 2001; Schollmeier 2001). Each individual computer of a peer stores a redundant part of a digital artifact, or even an entire copy of it. This means that even if one ‘node’ is lost, the digital artifact is preserved, since it is stored simultaneously on many other computers.

### Open licensing regime

In the theoretical foundation section, we discussed the issues inherent in the private innovation model and the role of intellectual property rights in this approach. Because of their specific properties, digital artifacts are hard to control, rendering the private innovation model even more problematic. Within the ecosystem, a licensing regime defines the legal options and restrictions as far as intellectual property is concerned, and, in our case, digital artifacts. Open licenses for software such as the GNU General Public License (Stallman 2002) or the Creative Commons licenses (Katz 2005) for content such as text, photos, or music allow unrestricted use and modification of existing digital artifacts, thus maximizing the benefit for sustainable development. In addition to condition number 3, representing technical openness, an open licensing regime ensures the legal openness of a digital artifact. Through its regulation, an open license facilitates the reuse and adaptation of previously created knowledge at no additional cost, preventing any unnecessary ‘reinventing of the wheel’. While the Open Definition (Open Knowledge 2015) clearly sets out the fundamental requirements of an open license, it is flexible if the derived work needs to be licensed under the same terms or if it can be integrated into proprietary digital artifacts. The effect when an open license requires derived digital artifacts to adopt the same license conditions is called “copyleft” (Mustonen 2003; de Laat 2005). This may hinder the use of such licensed software or other digital artifacts when users do not want their enhancements to be openly published. Therefore, many open licenses do not enforce their terms vis-à-vis derived work.

### Shared tacit knowledge

Skills and experience are necessary to use and in particular to advance digital artifacts (Nonaka and Konno 1998). Due also to the rapidly changing environment, the structures of digital artifacts need to be continuously adapted with respect to new interfaces, standards, and other technologies (Banker et al. 1998). Thus, tacit knowledge of digital artifacts is necessary to preserve and enhance their value within the ecosystem by means of socialization and externalization. Independence from single individuals or institutions reduces the risk of deterioration and abandoning of digital artifacts. Thus, shared tacit knowledge among many humans and organizations increases independence and longevity of such ecosystems. Communities of practice (Wenger 2004) as introduced above, as well as collective intelligence within peer production (Benkler et al. 2015) represent established forms of tacit knowledge-sharing within ecosystems.

### Participatory culture

Another aspect related to the notion of tacit knowledge-sharing is a stimulating environment, leading to contributions by the ecosystem. In open source communities, for example, Lakhani and von Hippel (2003) found that individuals contribute their time and skills for an open source project because they use the software for their own needs, because they enjoy programming, and because they want to boost their reputation. Such motivations indicate a community in which contributions are welcome and, thus, participation is part of the cultural rules and norms. Similarly, Benkler (2013) defined peer production as open creation and sharing performed by online groups, another setup in which participatory culture is required. Integrating knowledge and experience from various stakeholders demands effective quality control. Peer review processes are often applied to address this challenge (Viégas et al. 2007).

### Good governance

Nowadays, many digital artifacts are centrally controlled by a single corporation. In the interests of sustainable digital artifacts, governance ought to be shared among many stakeholders. To this end, an ecosystem should be organized with clear rules that apply to all participants. For example, open source developers in Debian and other communities have implemented strict rules on decision-making, collaboration, and communication (O’Mahony and Ferraro 2007). There are also clear guidelines within the Wikipedia community on how to edit content pages (Viégas et al. 2007). In the physical world, Ostrom’s (2005) work on commons-based governance similarly distributes the power among many, leading to good governance.

### Diversified funding

In the theoretical foundations, we discussed the economic dimension of digital artifacts and its implications for innovation. Some services related to the creation and use of digital artifacts may be provided by voluntary contributors, but others have to be generated by paid activities. Operating the servers, managing the platform with employees and taking care of administrative work may require substantial funding. Many digital artifacts are funded by a single organization and, therefore, depend on the existence of that organization. It seems to be less risky if financing is diversified among many stakeholders, since this reduces centralized control of a single entity, as well as the risk of conflicting interests. Crowd-funding is a common approach used by NGOs and startup companies to cover initial investment costs (Belleflamme et al. 2014). Recurring donations are used to cover operational costs of providing digital artifacts (Mary-Ann Russon 2015). In addition, it is common for a non-for-profit association or foundation to be set up in order to manage donations and provide operational services for the digital artifact (Baars and Jansen 2012; Riehle 2010). Among other tasks, such organizations manage the fair use of the financial resources received from its members, which can include corporations, governments, and universities.

### Contributing to sustainable development

The existence of digital artifacts, as well as their creation and use, may have manifold effects on sustainable development, both positive and negative. In order to better analyze the different contributions made by digital artifacts to sustainable development, a differentiated approach is valuable. Hilty and Aebischer (2015) suggest distinguishing between effects on three different levels. The “Life-Cycle Impacts” (Level 1) are direct effects of the use of hardware and other ICT-infrastructure. These consist of material resources and, therefore, are part of the problem of achieving sustainable development. The “Enabling Impacts” (Level 2) are indirect effects of the application of digital artifacts. These may lead to changes in production and consumption on the micro level. The changes may result, e.g. in optimized processes, which might save natural resources or help in recycling materials. The “Structural Impacts” (Level 3) are socio-economic effects of the use of IT-applications. These may lead to persistent changes on a structural and institutional level and, therefore, occur on a macro level. The effects on climate change of the distribution of information through digital media may result, e.g. in more consciousness in traveling by air or in supporting environmental and climate politics. The impacts of both Level 2 and Level 3 with respect to sustainable development can be positive, but may also be negative. To comply with our basic condition 10, the impacts of a digital artifact on those two levels need to be predominantly positive. Furthermore, these positive impacts should outweigh the negative effects of Level 1.

## Cases of sustainable digital artifacts and ecosystems

In our view, the ten basic conditions are key for the sustainability of digital artifacts and their ecosystems. However, we have to look into specific cases to validate whether these conditions hold in practice. Table 3 presents four cases that, in our opinion, illustrate how digital artifacts and their ecosystem are handled in specific projects: the Linux kernel development as an example of an open source project; Bitcoin as the most popular peer-to-peer open source cryptocurrency; the Wikipedia platform as an example of an open content initiative; and the Linking Open Drug Data (LODD) task force as an example of Linked Data technologies. These cases are well documented in the various papers referenced below. In Table 3, we integrate the results of the evaluations of the respective papers for the different projects. We have marked what we see as deficiencies in grey.

**Table 3:**
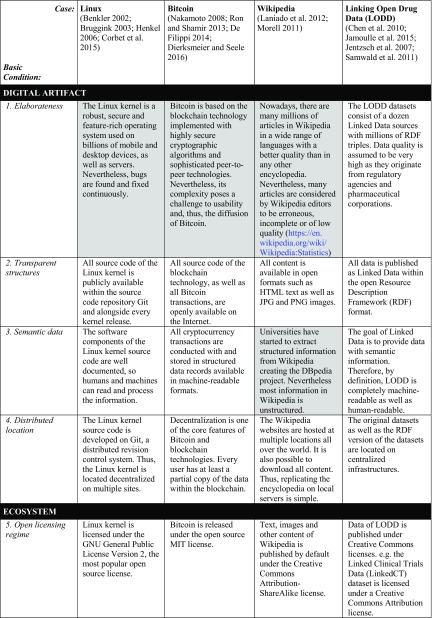

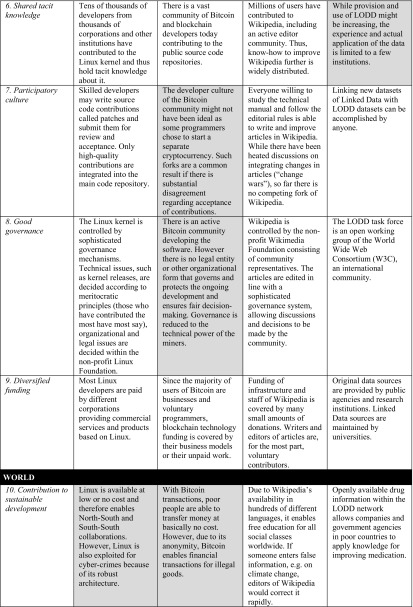
Cases of sustainable digital artifacts and their ecosystems

The basic conditions we have formulated are quite challenging and, therefore, difficult to achieve. In fact, many of today’s digital artifacts do not fulfill all (if any) of these criteria. Nevertheless, there are some digital artifacts and ecosystems that can be considered to at least partly achieve the basic conditions. While all four cases illustrate well the way in which sustainable digital artifacts and initiatives function, none of them fulfill all basic conditions completely. In the following, we discuss the conditions and the relationships between them.

## Discussion of the basic conditions

In our paper, we propose ten basic conditions for the sustainability of digital artifacts, their ecosystems, and the position of the ecosystem in the world as a whole. The four cases illustrate the role of the basic conditions in the context of various well-known open source or open data projects. The characteristics of each of the projects with respect to each of the basic conditions are outlined in Table 3.

The overview of the results shows that most of the basic conditions apply to the relevant cases. Some of the conditions are fulfilled for all of the cases: transparent structures, distributed location, open licensing regime, and diversified funding. The criteria met by at least three cases are semantic data, shared tacit knowledge, participatory culture, and good governance. Elaborateness and contribution to sustainable development are the only basic conditions not met by several cases. Of course, the compliance of the cases to some of the basic conditions is attributable to the choice of cases. This is particularly true of the open licensing regime: since we have chosen only ‘open projects’, all of them are subject to an open license. On the other hand, the rather negative assessment of elaborateness ought to be relativized since the projects concerned are highly complex and, thus, automatically more prone to a multitude of errors. The negative assessment of the contribution of this factor to sustainable development lies in the fact that digital artifacts can often be used for activities beneficial to sustainable development and also for detrimental purposes. Often, their mere existence does not pre-define their intended use. Furthermore, the analysis of the cases reveals how the basic conditions are interrelated to one another. They present a dynamic set of characteristics continuously influencing the sustainability of the digital artifacts and ecosystems, as the following explanations indicate.

### Elaborateness and participatory culture

The four cases illustrate how difficult it is to provide an elaborate digital artifact. Due to the continuously expanding requirements of the Linux kernel, it is basically impossible to provide flawless software. While Bitcoin has a robust technical foundation, use and integration of its technology is complex, making its usability a deficiency. The wide range of people involved in writing Wikipedia articles and the rapidly changing reality obviously makes it impossible to cover all topics in high-quality articles. Only the Linked Open Drug Data are assumed to be correct as it stems from regulatory agencies and pharmaceutical corporations subject to stringent controls. Elaborateness of dynamically changing digital artifacts will therefore remain a challenge in most contexts. However, if the basic conditions of the ecosystem are fulfilled, elaborateness of the digital artifact can be assumed to increase steadily as it is affected, e.g. by peer-review processes of a participatory culture. If culture invites the best contributors to participate, the digital artifact will steadily become more elaborate, thus increasing sustainability.

### Transparent structures and semantic data

The cases presented provide complete public access to their source code and data. Therefore, anyone with the requisite skills can study the technical structures and adapt them if necessary. However, open source and open data do not always come with semantic data. As the example of Wikipedia illustrates, only a small portion of the millions of pages is enhanced with semantic data. Most content consists of nothing more than formatted text without any semantics. Awareness of the value of semantic data is growing, which was what recently prompted the Wikimedia Foundation to start the Wikidata project, the aim of which is to build a complete linked open data repository (Vrandečić and Krötzsch 2014).

### Open licensing regime and diversified funding

Like many other open source and open content projects, the selected cases are also published under an open licensing regime. This allows anyone to take the digital artifact and develop their own version of it. Usually, this is not an efficient approach since it is only through collaboration with others that the digital artifact improves. However, if, due to failed governance, there is a serious conflict among stakeholders within the ecosystem, a community might split into a fork. This has happened several times in the Bitcoin community (Gandal and Halaburda 2014) but not with Linux and Wikipedia, where good governance has prevented separation of the communities thus far. Nevertheless, forking also happens in other open source projects (Nyman and Lindman 2013), particularly when funding is not diversified but is provided by a single corporation only. The external community might become frustrated if the single funding corporation focuses on commercialization rather than advancing the digital artifact. From this perspective, the Bitcoin case is somewhat of an exception, since it is a community-driven open source project that is still experiencing forking.

### Shared tacit knowledge and distributed location

The cases of Linux and Bitcoin illustrate how open source softwares and their communities fulfill the basic conditions for sustainability of digital artifacts and their ecosystem in several points. However, not every open source project complies with the conditions as fully as the Linux kernel or the Bitcoin community. While the open licensing regime and the transparent structure condition apply in all cases based on the definition of open source software, often tacit knowledge is not shared among several programmers. Many open source projects are developed by only one or a very small number of programmers leading to a high dependency on these persons (Krishnamurthy 2002). Moreover, the four cases presented are distributed on multiple locations as they can be considered mature digital artifacts. However, many other similar digital artifacts are organized less professionally and, thus, are available only on a single server, for example.

### Good governance and contribution to sustainable development

Ex ante, it is often hard to predict whether a digital artifact is beneficial to sustainable development or not . Often, the same digital artifact can simultaneously be used for contributions to sustainable development and support unsustainable behavior. The case of Bitcoin shows that the technology has the potential to reduce poverty, the amount of debt crisis, and inflation, but on the other hand enables shadow banking to buy weapons, drugs, and sex (Dierksmeier and Seele 2016). The cases of Linux and Wikipedia also illustrate this ambiguity. For example, Linux and Wikipedia can be used in the global south to facilitate a low-cost infrastructure (Linux) and provide access to education (Wikipedia), but they can be also used for unethical actions such as cyber-crime or learning how to build bombs. Openness and Transparency are important issues in this respect because these properties favor critique and self-regulation. Similar to the field of business ethics (Dierksmeier and Seele 2016), the moral ambiguity of digital artifacts is also a matter of perspective (deontological, teleological, utilitarian etc.). Therefore, good governance has to establish rules on how to overcome potentially unsustainable impacts of digital artifacts.

## Limitations and future research

Starting from the underlying assumption of the key role played by knowledge in the concept of sustainability, this paper explored how that knowledge needs to be handled in order to provide the greatest possible benefit to sustainable development. Due to the digitization of information, we focused on digital artifacts and the ecosystem in which they are embedded. The relationships between the digital artifacts and their ecosystem have been generalized as acts of creation and use. We consider those digital artifacts to be sustainable that are created and consistently adapted to the need of prospective or current users, and that are used as frequently as possible, wherever the digital artifact has a potential benefit to sustainable development. To achieve this goal, this paper examined ten basic conditions related to the digital artifacts, their ecosystems, and their embedding in the world at large. We used four cases to illustrate these basic conditions. This enabled us to demonstrate the extent to which these basic conditions are relevant for actual digital artifacts and information technology innovations.

However, there are several limitations to our work. Above all, our paper is intentionally conceptual. Despite having a sound foundation in the literature, the proposed ten basic conditions are tentative. The use of illustrative cases should not be misinterpreted as an empirical test. They show only that the basic conditions can be identified and are existent to a certain extent in some important and long-established projects. However, it cannot be concluded that these conditions are the reason why these projects have this standing. Furthermore, we have not validated the ten basic conditions with respect to their importance. We need sound empirical evidence to validate whether the proposed basic conditions are indeed causative for the sustainability of digital artifacts. Empirical research is, therefore, required before our model can be considered to be a reliable framework.

With respect to the stipulated basic conditions, we have tended towards the vision of an open collaborative innovation model. Our implicit understanding is that the development and use of knowledge is an inherently cooperative process in which we build new knowledge on top of the existing knowledge inherited from past generations. The circumstances under which and the extent to which the creation of digital artifacts within the more traditional private innovation model can also be classified as sustainable will need to be explored in greater detail.

In our paper, we generally assume that ensuring that digital artifacts are sustainable is the best way of tapping their potential to support sustainable development. However, we acknowledge that the use of digital artifacts may also be negative and detrimental to sustainable development. Therefore, we introduced basic condition 10 to enforce the consideration only of digital artifacts with predominantly positive impacts. While this basic condition may be criticized as being rather self-referential, it is vital for supporting sustainable development. By emphasizing principles like openness, transparency, and governance, we believe to have introduced favorable conditions so that digital artifacts with predominantly positive impacts on sustainable development will be created und used. However, the effect of these principles on ensuring the positive relationship between using digital artifacts and achieving sustainable development need to be elaborated in more depth.

Furthermore, we examined the benefits of sustainable digital artifacts without, however, focusing on any possible negative impacts of the use of technical infrastructure. As we have mentioned, digital artifacts are both material and immaterial. We need natural resources for the material part, i.e. the processing of data and its storage on hardware devices; this could have a potentially negative impact on sustainable development. We have neglected this aspect in our paper and, thus, implicitly assumed that, compared to the benefits of higher accessibility, the negative impact on the environment of the large-scale use of hardware is marginal in view of the advantages of not having to recreate the same knowledge over and over. This general assumption should be verified on a case-by-case basis.

Last, our discussion on the sustainability of digital artifacts and the surrounding ecosystem does not include the capability of individuals to participate in such an ecosystem. This depends on certain individual capabilities, sometimes referred to as digital literacy. One may assume that people need to be trained to be able to participate in digital ecosystems to achieve the desired impact of digital artifacts. Furthermore, there might be geographical and social limitations preventing people from participating fully in digital ecosystems, as discussed under the term ‘digital divide’. These more social factors empowering people to participate in digital ecosystems have also been excluded from this paper but are certainly worthy of closer attention.

Following the limitations, we outline subsequent issues, as well as some possible future research agenda addressing promising topics in relation to digital sustainability. First, empirical evidence has to be provided for the ten basic conditions. Therefore, the basic conditions will need to be operationalized in order to elaborate a measurement model. Additionally, such a measurement model would allow the importance of each of the ten basic conditions to be examined and furthermore provide evidence as to whether those conditions have a causal effect or not on the sustainability of digital artifacts. Second, non-sustainable digital artifacts should be analyzed to gain more insights into other possible basic conditions. Non-sustainable digital artifacts could be, e.g. failed software projects, where the source code is no longer available. Third, business models governing how organizations can best share knowledge and simultaneously make sufficient revenues to not endanger their own existence also need to be evaluated.

As this list of unanswered questions indicates, research on sustainability of digital artifacts is in its very early stages. We, therefore, propose that this area be advanced further by new theoretical and empirical research exploring how best to maximize the use of digitalization for the benefit of sustainable development, under the umbrella term of ‘digital sustainability’. This term is in line both with social sustainability (focus on society and people) and environmental sustainability (focus on the environment). Furthermore, we believe that the existing research on Green in IS and Green by IS (Esfahani et al. 2015) needs to be enhanced with research on the sustainability of digital artifacts (such as the core topic of this paper) to complete the puzzle of digital sustainability.
